# Supplementary material for: Overexpression of the Ubiquitin Specific Proteases USP43, USP41, USP27x and USP6 in Osteosarcoma Cell Lines: Inhibition of Osteosarcoma Tumor Growth and Lung Metastasis Development by the USP Antagonist PR619
Source: Cells. 2021 Aug 31;10(9):2268. doi: 10.3390/cells10092268 (PMC8464711; doi:10.3390/cells10092268)
Supplement: Supplementary file 1 [file cells-10-02268-s001.zip › cells-1276973-SI.pdf]

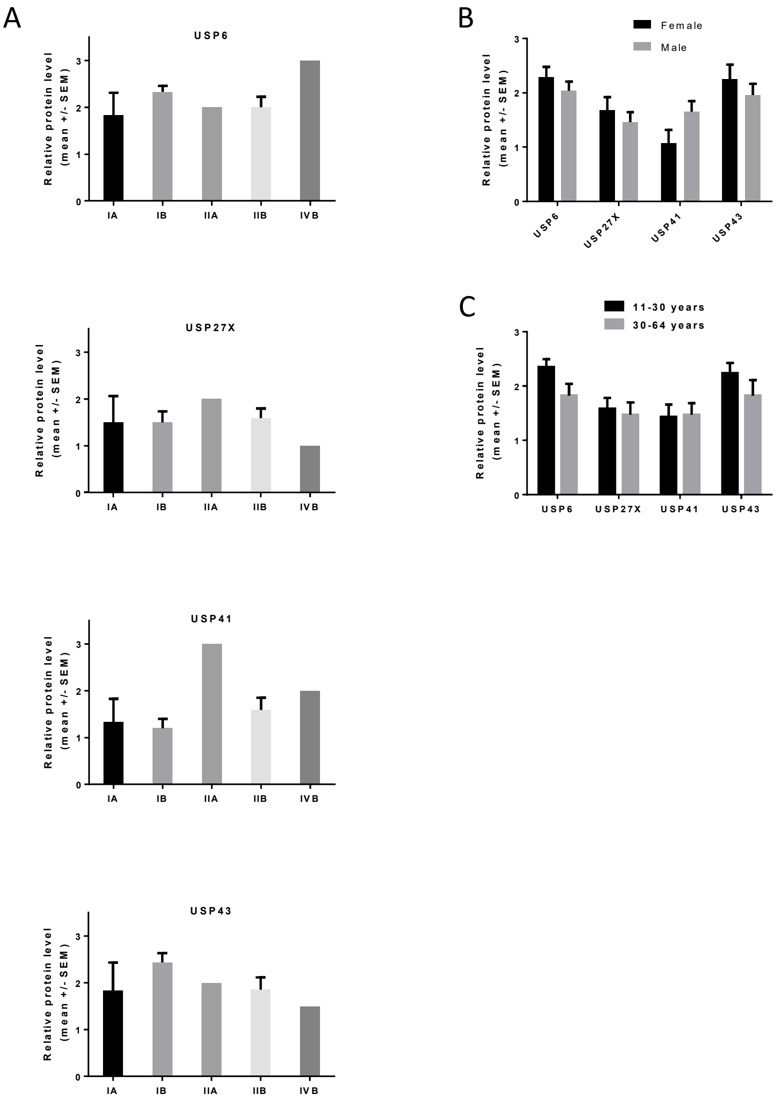

Figure S1: USPs expression as a function of disease stage, gender, or age of patients.
TMA samples were stained with USP43, USP41, USP27x, and USP6 antibodies as described in the materials and methods section, and in Figure 2. Staining intensity was scored relative to the most stained section of the slides. USPs expression ranking was evaluated as no expression, low expression, intermediate expression, and high expression. The expression ranking was finally done by giving a value for each expression intensity from 0 (no expression) to 3 (high expression). The relative expression level of USPs (mean +/- SEM) was given as a function of (A) disease stage (1A to IVB), (B) patient gender (female or male), and (C) patient age (two groups of equivalent size were defined, the first with patients aged from 11 to 30 years and the second with patients aged from 30 to 64 years).

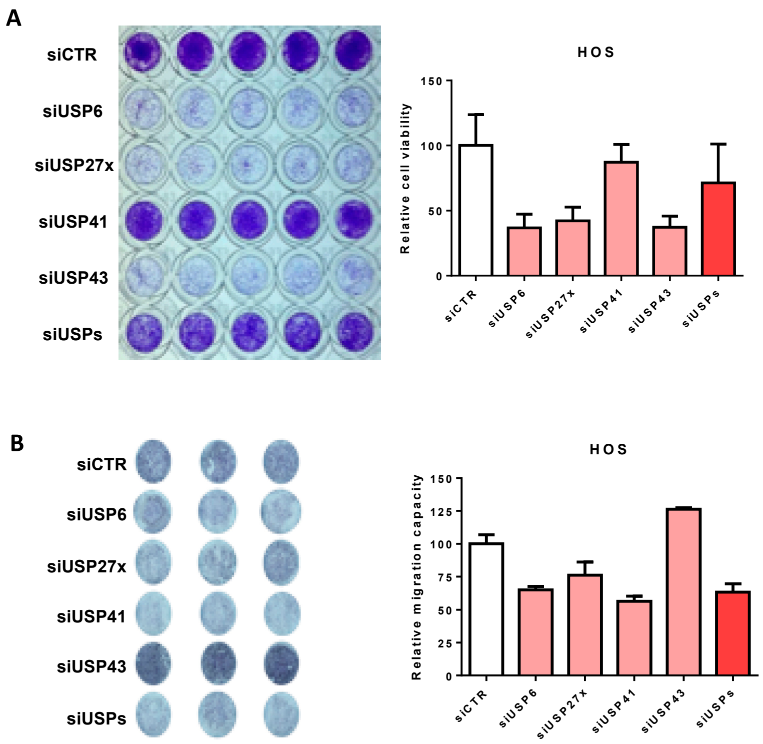

Figure S2: Decreased expression of USPs affects the viability of OS cells and their ability to migrate.
200000 HOS cells grown in 3.5 cm diameter plates are transfected with 30 nM of USPs siRNA (USP6, USP27X, USP41, and USP43), scrambled siRNA (CTR), or with all siRNA (siUSPs, 7,5 nM each) as indicated. A) 24 hours after transfection, 2000 cells were seeded in 96-well plates. Cell viability was assessed 48 hours later, as described in Materials and Methods, using crystal violet staining. Left panel: representative photograph after labeling of cells with crystal violet. Right panel: graph shows the relative cell viability after transfection of cells with USPs siRNAs compared to cells transfected with control siRNAs (mean ± SD of two experiments each performed in quintuplets). B) 48 hours after transfection, 60000 cells were seeded in Boyden chambers as described in the Materials and Methods section. Cell migration was assessed 8 hours later. Left panel: representative photograph after labeling of cells with crystal violet. Right panel: graph shows relative cell migration after transfection of cells with USPs siRNAs compared to cells transfected with control siRNAs (mean ± SD from a representative of two experiments each performed in triplicate).
